# The small molecule inhibitor anle145c thermodynamically traps human islet amyloid peptide in the form of non-cytotoxic oligomers

**DOI:** 10.1038/s41598-019-54919-z

**Published:** 2019-12-13

**Authors:** Manikam S. Saravanan, Sergey Ryazanov, Andrei Leonov, Janine Nicolai, Patrique Praest, Armin Giese, Roland Winter, Lucie Khemtemourian, Christian Griesinger, J. Antoinette Killian

**Affiliations:** 1 Membrane Biochemistry and Biophysics, Bijvoet Center for Biomolecular Research, Utrecht University, Padualaan 8, 3584 CH Utrecht, The Netherlands; 2 NMR based structural biology, MPI for Biophysical Chemistry, Am Fassberg 11, 37077 Göttingen, Germany; 3 DFG Research Center Nanoscale Microscopy and Molecular Physiology of the Brain, Göttingen, Germany; 4 Cluster of Excellence “Multiscale Bioimaging: from Molecular Machines to Networks of Excitable Cells” (MBExC), University of Göttingen, Göttingen, Germany; 5 Physical Chemistry I - Biophysical Chemistry, TU Dortmund University, Faculty of Chemistry and Chemical Biology, Otto Hahn Str. 4a, D-44221 Dortmund, Germany; 6 Medical Microbiology, University Medical Center Utrecht, 3684CX Utrecht, The Netherlands; 7 Zentrum für Neuropathologie und Prionforschung, Ludwig-Maximilians - University München, München, Germany; 8 Sorbonne Université, Ecole Normale Supérieure, PSL University, CNRS, Laboratoire des Biomolécules (LBM), 4 place Jussieu, F-75005 Paris, France; 9 Present Address: Institute of Chemistry & Biology of Membranes & Nanoobjects (CBMN), CNRS UMR5248, University of Bordeaux, Bordeaux INP, allée Geoffroy St-Hilaire, 33600 Pessac, France

**Keywords:** Prions, Intrinsically disordered proteins

## Abstract

Type 2 diabetes (T2DM) is associated with aggregation of the human islet amyloid polypeptide (hIAPP) into cytotoxic amyloid species. Here we tested the effect of a diphenylpyrazole (DPP)-derived small molecule inhibitor, anle145c, on cytotoxicity and on aggregation properties of hIAPP. We demonstrate that incubation of hIAPP with the inhibitor yields ~10 nm-sized non-toxic oligomers, independent of the initial aggregation state of hIAPP. This suggests that anle145c has a special mode of action in which anle145c-stabilized oligomers act as a thermodynamic sink for the preferred aggregation state of hIAPP and anle145c. We also demonstrate that the inhibitor acts in a very efficient manner, with sub-stoichiometric concentrations of anle145c being sufficient to (i) inhibit hIAPP-induced death of INS-1E cells, (ii) prevent hIAPP fibril formation in solution, and (iii) convert preformed hIAPP fibrils into non-toxic oligomers. Together, these results indicate that anle145c is a promising candidate for inhibition of amyloid formation in T2DM.

## Introduction

Misfolding of proteins and aggregation into amyloid structures is associated with a number of diseases, including type 2 diabetes (T2DM), Parkinson’s disease and Alzheimer’s disease^1–4^. These so-called amyloid diseases in general have the hallmark of an age-related pathological effect. However, T2DM stands apart in that it is a common metabolic disease, linked to life style and obesity^5–7^, that increasingly affects people even at an early age^7–9^. Its prevalence around the world is beginning to take on epidemic proportions and T2DM thereby represents a major health problem in society^7,10^.

A prominent pathological event in T2DM is believed to be the formation of toxic amyloid species by a 37 amino acid protein known as human islet amyloid polypeptide (hIAPP)^11–13^. hIAPP is a hormone that is co-secreted with insulin in the β-cells in the islets of Langerhans in the pancreas. Under pathological conditions typical for T2DM, hIAPP is produced at relatively high concentrations, thereby promoting aggregation and contributing to pancreatic cell death^14,15^. The aggregation process furthermore can be affected by factors such as the presence of metal ions, insulin, sulfated glycans and membranes^16–25^, thereby complicating interpretation of *in vivo* studies on a molecular level. Nevertheless, an important role for hIAPP in T2DM is underscored by the observations that a high prevalence of hIAPP aggregates is found in humans with T2DM^5,11,26^ and that for species whose IAPP cannot form fibrils it has been reported that they do not develop T2DM characterized by islet amyloid deposits^27–29^.

Inhibition of hIAPP amyloid formation is considered to be an effective strategy to help combat T2DM. Several natural small molecule amyloid inhibitor compounds, such as epigallocatechin gallate (EGCG), resveratrol and curcumin, as well as a variety of synthetic inhibitors have been shown to be able to inhibit hIAPP fibrillation or to reduce the cytotoxic activity of hIAPP^4,30–39^. However, most of these inhibitors were found to act efficiently only at relatively high concentrations.

Recently, a family of di-phenyl pyrazole (DPP) amyloid inhibitors has been developed that act as promising oligomer modulators to combat amyloid diseases^40^. Notably, the lead compound “anle138b” was found to inhibit protein aggregation in case of prion disease, Parkinson’s disease (PD), Alzheimer’s disease (AD), Multiple System Atrophy (MSA) and Creutzfeldt-Jakob disease (CJD) *in vitro* and *in vivo*^40–47^. For none of the DPP compounds, the effect on hIAPP has been tested. Yet hIAPP is an interesting target, because it forms oligomers that have some features that are different from those of other well-studied amyloid forming proteins^48^.

Here we set out to elucidate whether DPP compounds can be effective against aggregation of hIAPP. For this initial study, we selected the DPP-derived small-molecule inhibitor anle145c because it structurally resembles the very lipophilic lead compound anle138b and is likewise an efficient inhibitor of amyloid aggregation. Anle145c also has the advantage of being more water soluble than anle138b with a solubility between ~50 to 100 µM compared to ~0.2 µM for anle138b^40^. Therefore, artefacts due to aggregation of the inhibitor in the aqueous environment are avoided and the interaction of anle145c with hIAPP can easily and systematically be studied at concentrations that would not be feasible for anle138b.

Our findings indicate that the molecule efficiently inhibits both aggregation and cytotoxicity by hIAPP at sub-stoichiometric concentrations and in addition is able to convert mature amyloid fibrils into non-toxic anle145c-stabilized oligomers by stabilizing non-toxic oligomers thermodynamically. The results suggest that anle145c is a promising candidate to inhibit protein aggregation in case of T2DM.

## Results

### hIAPP and anle145c

**Figure 1:**
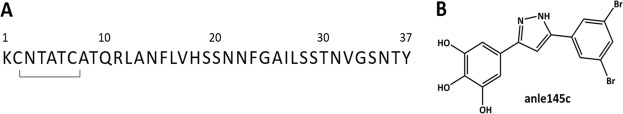
(**A**) Primary structure of hIAPP. The peptide has a disulfide bond between Cys2 and Cys7 and an amidated C-terminus. (**B**) Chemical structure of the DPP derivative anle145c.

In this study we investigated the interaction between hIAPP and the small-molecule inhibitor anle145c. Figure 1 shows the structures of these molecules.

### Anle145c protects cells from hIAPP-induced toxicity

The ability of anle145c to protect living cells from hIAPP-induced cytotoxicity was tested using insulinoma (INS-1E) cells from a rat pancreatic beta cell line. These cells closely resemble the insulin-producing beta-cells in the pancreas that are known to become damaged under conditions of T2DM^15^, most likely as a consequence of hIAPP aggregation.

**Figure 2:**
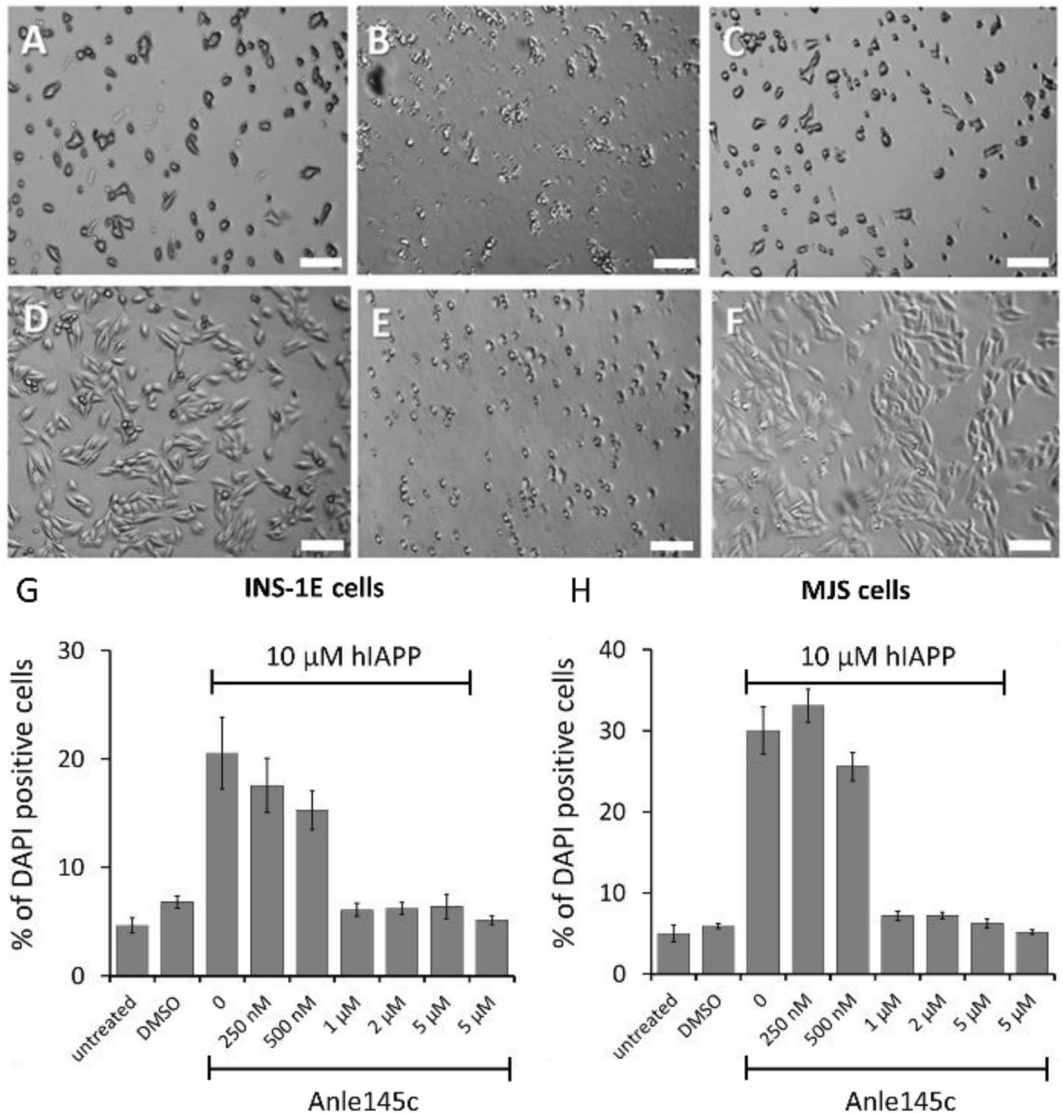
Effect of hIAPP and anle145c on INS-1E and MJS cells. Top: Microscopy images of INS-1E cells 6 h after incubation showing (**A**) untreated cells, (**B**) cells incubated with 10 µM hIAPP, and (**C**) cells incubated with 1 µM anle145c and 10 µM of hIAPP. Middle: Microscopy images of MJS cells 24 h after incubation showing (**D**) untreated cells, (**E**) cells incubated with 10 µM hIAPP, and (**F**) cells incubated with 1 µM anle145c and 10 µM of hIAPP. The scale bars represent 175 µm. Bottom: Quantification of the percentage of dead cells after 24 h of incubation, revealed by DAPI-staining for INS-1E cells (**G**) and MJS cells (**H**). Both panels show from left to right untreated and DMSO treated cells, followed by 10 µM hIAPP in the presence of different concentrations of anle145c, and 5 µM anle145c in the absence of hIAPP as indicated. All samples, except for the untreated cells, contained the same final DMSO concentration (5 vol %). The error bars represent the standard error from two independent experiments.

As shown by the micrograph in Fig. 2A, untreated INS-1E cells show a normal phenotype for adherent cells. Most of the cells are viable and attached to the cell culture dish. By contrast, the hIAPP-treated cells seem to have an impaired cell growth. Upon incubation with hIAPP a change in cell-morphology is observed and most of the cells are detached, indicating a considerable amount of cell-death (Fig. 2B). However, when a low concentration of anle145c is added prior to hIAPP, the cells again grow with a morphology similar as when no hIAPP is present (Fig. 2C). Flow cytometry based DAPI staining (Fig. 2G) confirms hIAPP-induced cell death and demonstrates that the inhibitor can rescue cell viability. A concentration of 1 µM anle145c, corresponding to a 1/10 molar ratio of inhibitor to hIAPP already is sufficient to yield a maximum rescue, while further increasing the anle145c concentration does not cause further inhibition of hIAPP-induced cell death. In addition, the results indicate that the concentration of DMSO, present in all samples containing either anle145c or hIAPP or both, does not cause substantial cell death and that also the inhibitor itself is not cytotoxic.

As an independent control model, we used cells from a human melanoma cell line (MJS cells), for which hIAPP is toxic. As shown in Fig. 2D, these cells have a distinctly different morphology from INS-1E cells. Nevertheless, for MJS cells similar results were obtained, i.e. a considerable amount of cell death upon addition of hIAPP (Fig. 2E,H), increased viability in the presence of low concentrations of inhibitor (Fig. 2F,H) and no apparent cytotoxicity of the inhibitor itself.

### Anle145c inhibits hIAPP fibrillation in solution by acting on late oligomers

To gain insight into the molecular nature of the interaction between hIAPP and anle145c, we next studied the aggregation behavior of hIAPP in the absence and presence of the inhibitor.

**Figure 3:**
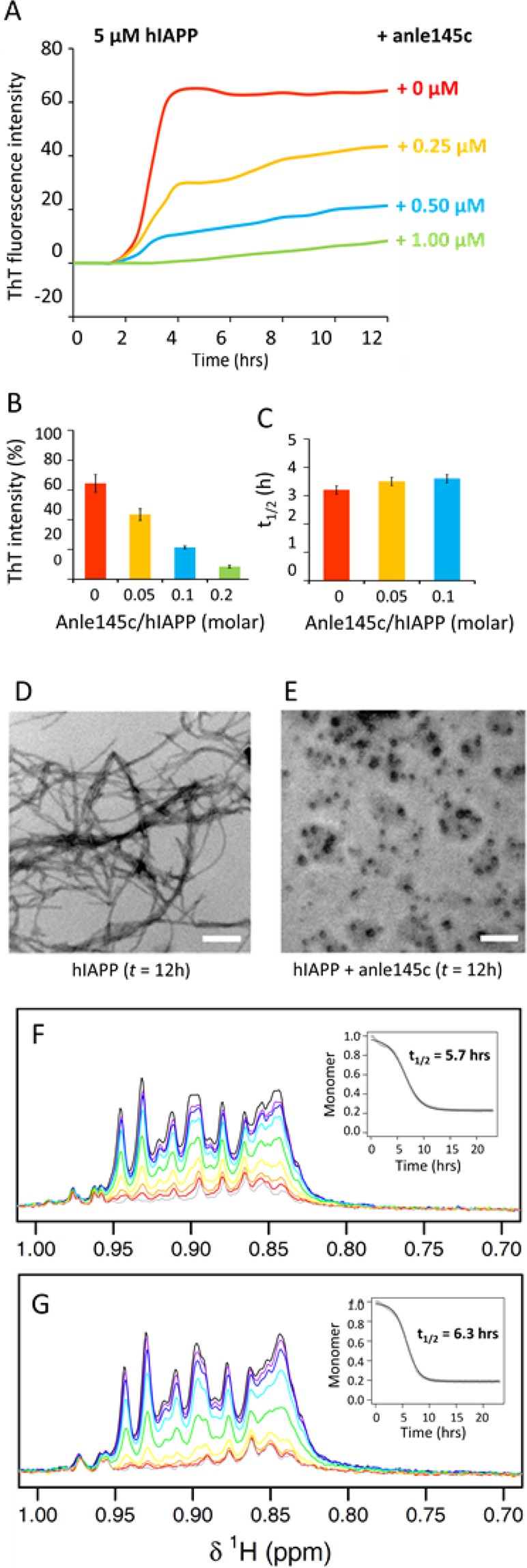
Effect of anle145c on hIAPP fibrillation in solution. (**A**) Kinetics of hIAPP fibrillation in the absence (red) and presence of inhibitor at different anle145c/hIAPP molar ratios as indicated in the figure (**B**) average ThT intensities after 12 hours of incubation and (**C**) average midpoints (t_1/2_) of the sigmoidal transition in the presence of different concentrations of anle145c. The error bars represent the standard deviation from two independent ThT experiments, each carried out in triplicate. (**D**) Negative stain TEM image of hIAPP after 12 h incubation in the absence of anle145c and (**E**) in the presence of anle145c at a 1/5 molar ratio of anle145c to hIAPP (white scale bars represent 500 nm). (**F**) ^1^H NMR spectra of the aliphatic region of hIAPP in time in the absence and (**G**) in the presence of anle145c at a 1/5 molar ratio of anle145c to hIAPP. The insert shows the integrated intensity of the peaks as function of aggregation time, corresponding to depletion of monomers and possibly of small, mobile oligomers.

The aggregation kinetics of hIAPP were followed using a ThT binding assay. ThT is a widely used fluorescent dye that does not bind to monomeric hIAPP, but shows enhanced fluorescence upon binding to amyloid structures^49^. As shown in Fig. 3A, incubation of hIAPP in buffer results in an initial lag phase of ~3 h, followed by a steep increase in ThT fluorescence intensity (growth phase), until after ~6 h a saturation level is reached (plateau phase). The presence of anle145c already at very low concentrations results in a considerable decrease in fluorescence intensity of the plateau region until at a 1/5 molar ratio of anle145c to hIAPP no fluorescence intensity increase can be detected anymore (Fig. 3B). Remarkably, the lag time of fibril formation seems hardly affected by the inhibition (Fig. 3C), suggesting that anle145c may act on late oligomers that are present just prior to fibril formation, thereby excluding them from participation in the fibrillation process. However, an alternative explanation could be that the inhibitor competes with ThT for binding on the fibrils, thereby reducing the ThT intensity^50^.

To discriminate between these possibilities, we performed negative stain TEM. As shown in Fig. 3D, hIAPP forms mature amyloid fibrils after 12 hours of incubation, whereas no fibril formation can be observed for hIAPP in the presence of anle145c at a 1/5 molar ratio of inhibitor to hIAPP (Fig. 3E). Instead, small non-fibril complexes are formed, confirming full inhibition of fibril formation at sub-stoichiometric concentrations of inhibitor.

If the inhibitor indeed acts on late oligomers, it should not significantly affect the kinetics of monomer depletion during aggregation. This was tested by ^1^H NMR on hIAPP in the absence and presence of anle145c as shown in Fig. 3F,G for the aliphatic region. In both cases a reduction of intensity over time is observed, due to depletion of monomers during amyloid formation and to the potential depletion of small oligomers that are still sufficiently small and mobile to contribute to the NMR signal^51^. Quantification of the signal intensity shows that the kinetics of monomer and small oligomer depletion indeed are similar with a t_1/2_ value of 5.7 hours in both cases. The data also indicate that hIAPP monomers are consumed almost completely. For the aromatic region no discernable signals were left (Supplementary Fig. S1), and hence it can be concluded that at most only a negligible amount of hIAPP monomers remain in solution.

### Biophysical properties of the anle145c-stabilized oligomeric complexes

The nature of the different hIAPP species formed upon incubation of hIAPP in the absence and presence of anle145c was further characterized by different biophysical approaches.

**Figure 4:**
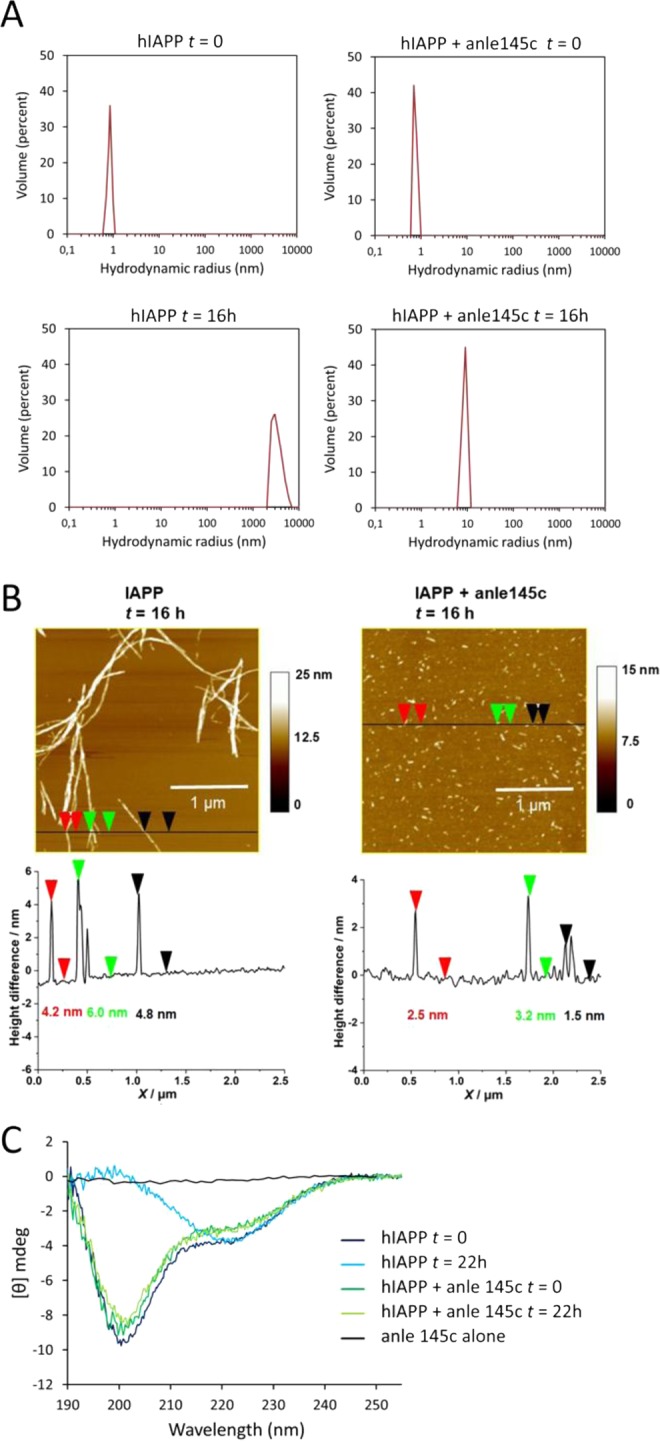
Characterization of hIAPP aggregates in the absence and presence of anle145c at a 1/5 molar ratio of anle145c to hIAPP. (**A**) DLS results showing the size distribution of hIAPP (left panel) and of hIAPP in the presence of anle145c (right panel) at time t = 0 and after 16 h incubation, as indicated in the figure. (**B**) AFM results showing a micrograph (top) and section analysis (bottom) of hIAPP (left panel) and of hIAPP in the presence of anle145c (right panel) after 16 h of incubation. (**C**) CD spectra of hIAPP in solution in the absence (blue) and presence (green) of anle145c immediately upon addition (dark lines) and after 22 h of incubation (light lines). The spectrum of anle145c in buffer is also shown (black).

The size of the particles was studied by dynamic light scattering (DLS). Figure 4A shows that freshly dissolved monomeric hIAPP in buffer has a hydrodynamic radius (R_H_) of ~1 nm. After 16 h incubation, only very large particles are observed of ~2–10 µm, consistent with the formation of amyloid fibrils. When freshly dissolved hIAPP is incubated in the presence of the inhibitor at a sub-stoichiometric ratio of anle145c to hIAPP of 1/5, the initial R_H_ is again ~1 nm. However, in this case after incubation only a relatively small increase in R_H_ is observed to around 10 nm.

The oligomeric species were further characterized by atomic force microscopy (AFM). As shown in Fig. 4B, hIAPP on its own forms fibrils with a length of several µm (left, top), while in the presence of inhibitor only small particles are observed (right, top). Individual height profiles obtained by section analysis showed a height of ~4–6 nm for the fibrils (left, bottom) and a height of approximately 1–3 nm for the particles in the presence of inhibitor.

Finally, the effect of anle145c on the conformational behavior of hIAPP was monitored using CD spectroscopy (Fig. 4C). CD spectra of freshly dissolved hIAPP in the absence and presence of anle145c display a peak with negative ellipticity at 200 nm, indicating that the peptide adopts mostly a random coil structure. After incubation the conformation of hIAPP in the absence of inhibitor changes to a pattern with a minimum around 220 nm, indicative of β-sheet formation, whereas no change is observed for hIAPP in the presence of a sub-stoichiometric ratio of anle145c to hIAPP of 1/5. These results suggest that anle145c either binds to oligomeric structures in which hIAPP has a similar conformation as freshly dissolved hIAPP, or that hIAPP oligomeric structures adopt such a conformation upon binding of the inhibitor, thereby effectively preventing it from forming β-sheet like fibrils.

### Anle145c converts hIAPP amyloid fibrils into non-toxic oligomeric structures

**Figure 5:**
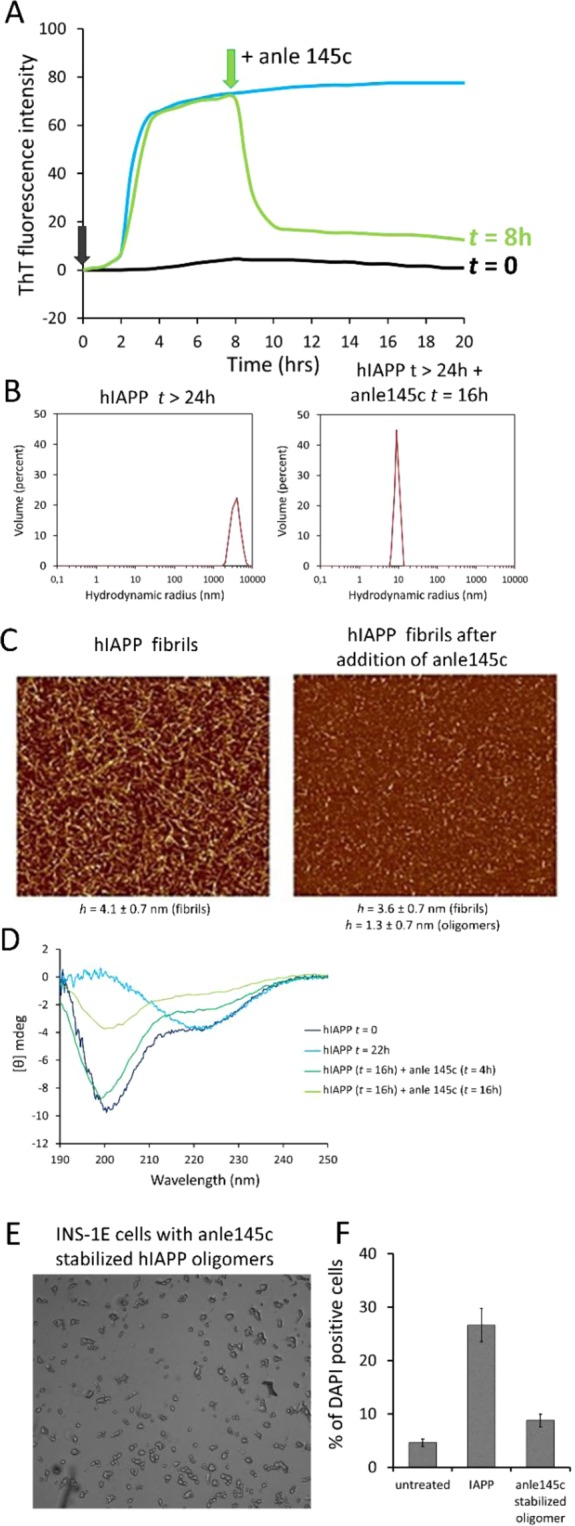
Effects of addition of anle145c to mature hIAPP fibrils at a 1/5 molar ratio of anle145c to hIAPP. (**A**) Kinetics of hIAPP fibril formation in the absence of anle145c (black) and upon addition of anle145c at t = 0 (blue) and at t = 8 h (green). (**B**) DLS showing the size distribution of fully formed hIAPP amyloid fibrils before addition of anle145c (top) and 16 h after addition of anle145c (bottom). (**C**) AFM of hIAPP after 16 h incubation, showing fully formed hIAPP fibrils (left) and of the same fibril containing sample after additional incubation with anle145c for 23 h (right). (**D**) CD spectrum of freshly incubated IAPP (dark blue), of hIAPP fibrils (light blue) and of hIAPP fibrils upon incubation with anle145c at time points: t = 4 h (light green) and t = 16 h (dark green). (**E**) Microscopy image of INS-1E cell after addition of anle145c-stabilized hIAPP oligomers derived by incubation of hIAPP fibrils with anle145c for 16 h (scale 200 μm). (**F**) Quantification of the percentage of dead cells 24 h after addition of hIAPP (left) and 24 h after addition of anle145-stabilized oligomers derived from hIAPP fibrils that were incubated with anle145c (right). The error bars represent the standard error from two independent experiments.

An important question is whether anle145c is able to act on grown fibrils, and, if so, what kind of structures are formed. As shown in Fig. 5A, addition of anle145c to hIAPP fibrils, again at a sub-stoichiometric concentration, results in a rapid decrease of ThT intensity, suggesting that the hIAPP fibrils undergo remodeling and are converted to smaller structures, that do not bind ThT. This was confirmed by EM (Fig. S2). Importantly, DLS measurements (Fig. 5B) and AFM measurements (Fig. 5C) show that the particle sizes obtained after starting from fibrils are similar to those obtained when the inhibitor is co-incubated with hIAPP monomers (Fig. 4A,B). DLS and AFM measurements in which anle145c was added at different times of incubation (Supplementary Figs. S3 and S4) further indicated that the final oligomeric state is independent of the starting aggregation state of hIAPP (Supplementary Tables S1 and S2). If this is the case, then anle145c should also be able to convert the β-sheet structures in hIAPP fibrils back into a mostly random coil conformation. CD measurements (Fig. 5D) of hIAPP fibrils upon addition of anle145c at different incubation times show that the conformation of hIAPP indeed reverts towards the conformation that was observed upon co-incubation of hIAPP monomers with the inhibitor (Fig. 4C).

The anle145c-stabilized oligomers can only be relevant *in vivo* if they are non-toxic to cells. Thus, the effect of the oligomers was tested on INS-1E cell lines. The results, as illustrated in Fig. 5E and quantified in Fig. 5F, show that the cells remain mostly viable when they are incubated with anle145c-stabilized oligomers derived from incubation of hIAPP fibrils, similar to the situation when monomeric hIAPP is added to cells in the presence of anle145c (Fig. 2F,G).

Together, the results obtained from addition of anle145c to hIAPP at different time-points of incubation indicate that anle145c-stabilized oligomers form a thermodynamic sink for the preferred aggregation state of hIAPP in the presence of anle145c and that these oligomers are non-cytotoxic.

## Discussion

Here we investigated the interaction between the DPP-derived small molecule inhibitor anle145c and hIAPP in solution and we investigated the effect of the inhibitor on hIAPP-induced cytotoxicity. We will first discuss the situation in solution.

We found that anle145c is a very efficient inhibitor of hIAPP aggregation in solution, with sub-stoichiometric concentrations of anle145c being sufficient for full inhibition of fibril formation. Instead of fibrils, oligomeric species were formed, which we call “anle145c-stabilized oligomers”. The size distributions as observed by DLS, EM and AFM experiments were consistent with oligomers of ~10 nm. The secondary structure of hIAPP in this oligomeric complex with anle145c was mostly random coil, similar to that of freshly dissolved monomeric hIAPP, but also similar to toxic as well as non-toxic oligomers^48^.

^1^H NMR experiments indicated that anle145c does not recruit monomeric hIAPP species or small oligomers, since its presence does not seem to affect the half-time of monomer depletion. Moreover, recruitment of monomeric species would effectively decrease the hIAPP monomer concentration, leading to an increase in lag-time of fibril formation^51–53^, which was not observed by ThT measurements. Instead, our finding that the lag time for fibril formation is independent of the anle145c concentration suggests that anle145c acts on late oligomeric species that are present just prior to fibril formation. This would then prevent them from becoming part of the growing fibrils, but would not interfere with the process of fibril formation itself.

It has been proposed that hIAPP oligomers undergo “activation”, *i.e*. a transition with a high energy barrier, from an oligomer in a random coil organization to an ordered β-sheet oligomer, which then can grow into fibrils^54^. Possibly, anle145c prevents this “activation” step by interacting with oligomeric species and allowing the formation of a thermodynamically more favorable state. This state then would consist of anle-stabilized hIAPP oligomers, in which hIAPP remains in a mostly random coil conformation.

Consistent with being the thermodynamically preferred species, anle145c-stabilized oligomers could not only be obtained by incubation with monomeric hIAPP species, they also could be derived by incubation with fibrils. Both oligomer populations were found to behave very similar, in the sense that they have a similar size distribution and conformation and they are non-toxic to cells. Moreover, addition of anle145c to hIAPP populations at different times of incubation suggested that formation of anle145c-stabilized oligomers is controlled by thermodynamics, *i.e*. no matter what the initial population distribution of hIAPP aggregates is, anle145c-stabilized oligomers will be obtained with similar final size distributions.

**Figure 6:**
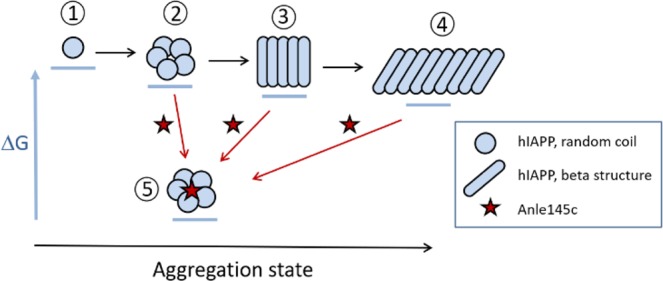
Model for the interaction of anle145c with hIAPP. Thermodynamics are qualitatively represented by the horizontal bars under the different aggregate states as differences in ΔG and kinetics are represented by arrows. The aggregation state is qualitatively represented on the horizontal axis. In the absence of anle145c (black arrows), hIAPP molecules in random coil conformation (1) interact with each other to form oligomers (2). In time the nature of these oligomers changes and β-sheet structures will be formed (3) that eventually will grow into mature amyloid fibrils (4) which are considered to be the thermodynamically most stable form. When hIAPP is incubated in the presence of anle145c (red arrows) it interacts with (late) hIAPP oligomers to form stable, non-toxic anle145c-stabilized oligomers (5), where the oligomers retain a mostly random coil configuration. Similar non-toxic anle145c-stabilized oligomers are obtained when anle145c is added to grown fibrils or to hIAPP aggregates formed at other time points, indicating that the anle145c-stabilized oligomers act as a thermodynamic sink. The exact pathway by which these anle145c-stabilized oligomers are formed is not known.

The exact pathways for fibril formation of hIAPP are not known but they are likely to involve many different potential oligomeric intermediates^48,55,56^. In Fig. 6, we present a simplified model to summarize the main effects of anle145c on hIAPP in solution. Notably, the stabilization of smaller non-toxic oligomers has also been observed *in vivo* for α-synuclein with the related anle138b^40,46^. Both observations are consistent with the *in vitro* design principle of the DPP compounds, which is conversion of larger into smaller oligomers^40^. Nevertheless, the effect of anle145c on hIAPP is remarkable for two reasons.

First, we used the more water-soluble variant of the lead compound anle138b, which has not been extensively tested so far for its ability to inhibit amyloid formation. Rather surprisingly, the results in this study show that anle145c is perhaps the most effective inhibitor for hIAPP fibril formation reported thus far. Indeed, at substoichiometric concentrations of anle145c it was found that (i) fibril formation is prevented, (ii) hIAPP-induced cell death is inhibited and (iii) fibrils are converted into non-toxic anle145c-stabilized oligomers. To our knowledge this is the first time that such a high efficiency for an hIAPP inhibitor compound is reported for all three effects. Anle145c furthermore appears to act in an unprecedented way by trapping hIAPP in a thermodynamic sink in the form of non-toxic anle145c-stabilized oligomers.

The second reason why the effects are remarkable, is that *a priori* one could not have made the assumption that DPP compounds would be effective against hIAPP toxic oligomers. This is because hIAPP oligomers have some features that are different from those of other well-studied amyloid forming proteins, such as α-synuclein^57^, i.e. they do not exhibit extensive secondary structure and they do not expose hydrophobic surfaces that allow them to bind hydrophobic molecules such as 1-anilinonaphtalene-8-sulphonic acid (ANS)^48^. The efficient inhibition of aggregation that was observed in the presence of anle145c, thus highlights the general efficiency of DPP compounds against amyloid aggregation.

In cells, we observed efficient inhibition of the toxic effects of hIAPP at even smaller sub-stoichiometric ratios than those required for inhibition of hIAPP aggregation in solution. This is not uncommon, as for inhibitor compounds where comparisons were made, higher as well as lower concentrations were found to be required in cells than in solution^31,32,58,59^. Importantly, the higher efficiency of anle145c in cells further increases the potential of this compound as an inhibitor to prevent hIAPP islet amyloid formation and thereby combat T2DM.

In conclusion, this study adds a highly promising inhibitor, anle145c, to the existing array of potential inhibitors of hIAPP amyloid formation and it adds T2DM as one more amyloid disease that may be targeted by DPP compounds. The results thus highlight the potential of DPP-derived compounds as small molecule oligomer modulators for amyloid diseases in general.

## Experimental Procedures

### Materials

Human hIAPP (amino acid sequence: KCNTATCATQRLANFLVHSSNNFGAILSSTNVGSNTY including disulfide bond and amidated C-terminus) was obtained from Bachem AG (batch number 7001439). Thioflavin T (ThT) was obtained from Sigma Aldrich. Anle145c was supplied by the NMR-based structural biology department, (MPIbpc, Göttingen, Germany) and was synthesized as described previously^40^.

### Preparation of stock solutions of hIAPP

hIAPP stock solutions were prepared as described previously^60^. Briefly, hIAPP was dissolved in 1, 1, 1, 3, 3, 3-hexafluoro-2-propanol (HFIP) and incubated for 1 hour to eliminate any pre-existing supramolecular structures. HFIP was evaporated using nitrogen gas followed by 1 h in a vacuum desiccator. For the fluorescence, microscopy (TEM, AFM), DLS and cell viability assays, the resulting peptide film was dissolved in DMSO at 2 mM and incubated for one hour. If required, hIAPP was further diluted in DMSO to the desired concentration. For the NMR experiments, the peptide film was solubilized at a concentration of 50 µM in 10 mM phosphate buffer, pH 7.4 in D_2_O. For the CD experiments, the peptide film was solubilized at a concentration of 25 µM in 10 mM phosphate buffer, pH 7.4.

### Preparation of stock solutions of anle145c

Stock solutions of anle145c were prepared as 10 mM solutions in DMSO, or in DMSO-d_6_ for the NMR experiments.

### ThT fluorescence assay

Thioflavin-T binding fluorescence assays were performed using a Spectrafluor Tecan (Salzburg, Austria) plate reader as previously described^61^. The kinetics of fibril formation were measured by monitoring the fluorescence intensity increase upon binding of the fluorescent dye Thioflavin T (ThT) to fibrils. A plate reader and standard 96-wells flat-bottom black microtiter plates in combination with a 430 nm excitation filter and a 535 nm emission filter were used. First 2.5 µL of an anle145c solution in DMSO at the desired concentration was added to 195 µL of a buffer containing 10 µM of ThT, 10 mM Tris-HCl, 100 mM NaCl at pH 7.4. The ThT assay was then started by adding 2.5 µL of a 0.4 mM hIAPP solution in DMSO. The microtiter plate was shaken for 10 s directly after addition of all components, but not during the measurement. All ThT assays were performed on triplicate samples on different days and using different hIAPP stock solutions. The resulting curves were analyzed using ORIGIN program and fitted to a Boltzmann sigmoidal equation, where F_i_ and F_t_ are the initial and final fluorescence values. This fitting allows the estimation of kinetic parameters such as the time for which the fluorescence reaches 50% of its maximal intensity (t_1/2)._

$${\rm{F}}=\frac{({\rm{Fi}}-{\rm{Ff}})}{1+{{\rm{e}}}^{({\rm{t}}-{\rm{t}}1/2)/\tau }}+{\rm{Ff}}$$

### Transmission electron microscopy

TEM images were recorded using a Technai 12 electron microscope operating at 120 kV as previously described^61^. hIAPP in solution was incubated in the absence and presence of anle145c under the same conditions as in the Thioflavin T assay. Aliquots (20 μL) of this mixture were placed onto glow-discharged 300-mesh carbon-coated copper grids for 2 min, then the grids were blotted and dried. Grids were negatively-stained, as described previously^61^, with saturated uranyl acetate (2% w/v) for 45 s, after which they were blotted and dried. Grids were examined using a Technai 12 electron microscope operating at 120 kV.

### Cell cultures

INS-1E rat insulinoma cells were cultured in RPMI 16% FCS, 100 U/ml penicillin, 100 µg/ml streptomycin, 10 mM HEPES, 1 mM sodium pyruvate, 2.5 mM glucose and 50 µM β-mercapto-ethanol. The human melanoma cell line MelJuSo (MJS) was cultured in RPMI 1640 (Invitrogen) containing 10% FCS (PAA laboratories), 100 U/ml penicillin, 100 µg/ml streptomycin and 2 mM L-glutamine (complete medium). All cell lines were cultivated at 37 °C and 5% CO_2_.

### Flow cytometry based DAPI staining

Cells were seeded prior to incubation with hIAPP. hIAPP was added to the cells in the presence of varying concentrations of anle145c. The final concentration of hIAPP was 10 µM. After treatment the cells were thoroughly washed with PBS, trypsinized and transferred to 96-well plates. The cells were incubated for 10 min with DAPI (2 µg/ml) and washed three times to remove the staining solution. All samples were measured by flow cytometry using a FACS Canto II and analyzed using FlowJo V10 software.

### NMR spectroscopy

NMR experiments were recorded on a Bruker Avance III spectrometer operating at a ^1^H frequency of 500 MHz and equipped with a TCI cryoprobe (temperature 20 °C) as previously described^51^. Experiments were processed and analyzed with TopSpin program (Bruker). Measurements were carried out in 10 mM potassium phosphate buffer in D_2_O at pH 7.4. Peptide concentrations were 50 µM in the absence and in the presence of anle145c. The peptide film was dissolved in the phosphate buffer and immediately transferred in a Shigemi tube of 5 mm diameter, comprising a sample volume of 300 µL. The evolution in time of the peptide signal intensity was followed by recording one-dimensional ^1^H spectra until the peptide signal intensity decayed completely. Peptide ^1^H resonances (both in the aliphatic and in the amide/aromatic region) were then integrated and these values are plotted as a function of time, leading to a sigmoidal curve that can be fitted using R program by a Boltzmann sigmoidal equation.

### DLS analysis

DLS measurements of hIAPP in the absence and presence of varying concentrations of anle145c were performed at room temperature on a Zetasizer Nano ZS (Malvern Instruments, United Kingdom). Aliquots of 2.5 µL of an anle145c solution in DMSO at the desired concentration were added to 195 µL of a buffer containing 10 mM Tris-HCl, 100 mM NaCl at pH 7.4. Subsequently 2.5 µL of a 0.4 mM hIAPP solution in DMSO was added and the mixture was transferred to a low volume cuvette for measurements. Each measurement was an average of seven runs and took about 7 minutes to complete. The volume percentage was recorded, which describes the relative proportion of multiple components in the sample based on their mass or volume rather than based on their scattering (intensity). Processing of the data was automated through the Zetasizer software.

### AFM analysis

AFM measurements were performed at room temperature using scan rates between 1.0 and 1.5 Hz in the tapping mode on a MultiMode scanning probe microscope equipped with a Nano-Scope IIIa controller (Digital Instruments) using an E-scanner (scan size 15 mm-615 mm) and a MMMC cantilever holder (Veeco Instruments, Mannheim, Germany) equipped with a silicon SPM sensor (PPPNCHR, NanoAndMore, Wetzlar, Germany) as previously described^62^. hIAPP (50 µM) without and with anle145c (10 µM) was incubated for various time intervals in 10 mM Tris-HCl, 150 mM NaCl buffer solution and then deposited on freshly cleaved mica. The samples were dried with a gentle stream of nitrogen, rinsed with water, dried again with a stream of nitrogen and finally freeze-dried overnight, as described previously^61^. The dried samples were scanned in air with drive frequencies around 240 kHz and drive amplitudes between 15 and 379 mV. All height and amplitude images of sample regions were acquired with resolution of 5.1 Mpixels. For image analysis and processing, the software NanoScope version 5 (Veeco Instruments, Mannheim, Germany) was used.

### CD spectroscopy

CD spectra were measured on a Jasco 810 spectropolarimeter (Jasco Inc., Easton, MD). Measurements were carried out in cells of 0.1 cm path length at room temperature in 10 mM phosphate buffer at pH 7.4 of hIAPP at different times of incubation in the presence and absence of anle145c. Measurements were taken every 0.2 nm at a scan rate of 20 nm/min. Each spectrum reported is the average of five scans. The peptide concentration was 25 µM. anle145c was added at a 5/1 molar ratio of hIAPP to anle145c^61^.

### Abbreviations

hIAPP, human islet amyloid polypeptide; ThT, thioflavin; RH, hydrodynamic radius; DLS, dynamic light scattering; AFM, atomic force microscopy; EM, electron microscopy. T2DM, type 2 diabetes; DPP, diphenylpyrazole.

## Supplementary information

Supplementary Information

## Data Availability

The datasets generated during and/or analysed during the current study are available from the corresponding author on reasonable request.
