# Supplementary material for: The small molecule inhibitor anle145c thermodynamically traps human islet amyloid peptide in the form of non-cytotoxic oligomers
Source: Sci Rep. 2019 Dec 13;9:19023. doi: 10.1038/s41598-019-54919-z (PMC6911113; doi:10.1038/s41598-019-54919-z)
Supplement: Supplementary file 1 — Supplementary Information [file 41598_2019_54919_MOESM1_ESM.pdf]

The small molecule inhibitor anle145c thermodynamically traps human islet amyloid polypeptide in the form of non-cytotoxic oligomers

Manikam S Saravanan, Sergey Ryazanov, Andrei Leonov, Janine Nicolai, Patrique Praest, Armin Giese, Roland Winter, Lucie Khemtemourian*, Christian Griesinger*, J Antoinette Killian*

Table of contents

Supplementary tables 3

Table S1. Overall particle size distribution analyzed by DLS. 3

Table S2. Individual height distribution analyzed by AFM 3

Supplementary data 4

Figure S1 4

Figure S2 5

Figure S3 6

Figure S4 7

Supplementary tables

<u>Table S1.</u> Overall particle size distribution analyzed by DLS.
(based on the results from Fig.4A and Fig.S3)

Incubation time of hIAPP before anle145c addition	Total incubation time	Final particle size of hIAPP+anle145c
**0**	16 h	~ 10 nm
**15 min**	16 h	~ 10-12 nm
**1 h**	16 h	~ 12-50 nm
**4 h**	16 h	~ 20-50 nm
**16 h**	39 h	~ 10-20 nm

<u>Table S2.</u> Individual height distribution analyzed by AFM
(based on the results from Fig.S4)

Incubation time of hIAPP before anle145c addition	Total incubation time	Final height of oligomers
**15 min**	16 h	~ 2.7 ± 0.7 nm
**1 h**	21 h	~ 1.5 ± 0.7 nm
**4 h**	21 h	~ 1.4 ± 0.7 nm
**16 h**	39 h	~ 1.3 ± 0.7 nm

Supplementary data

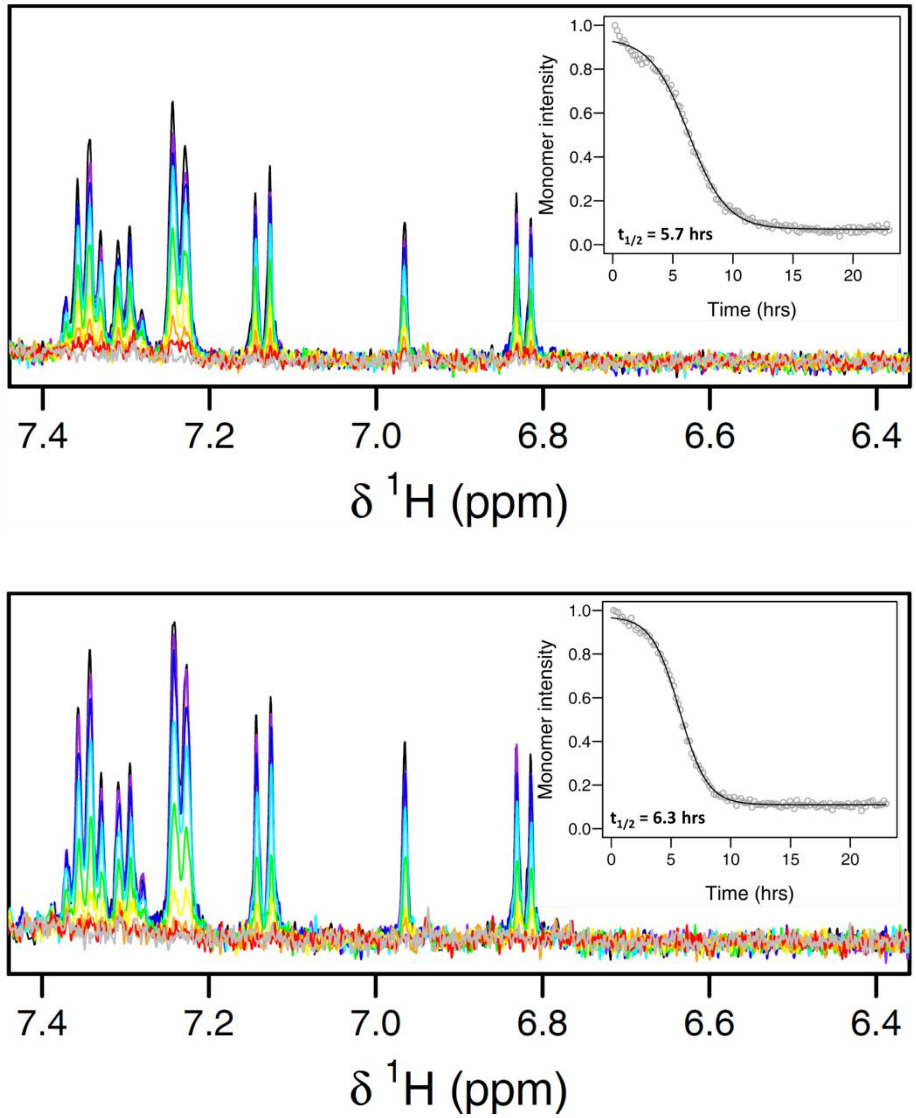

Figure S1. ^{1}H NMR spectra of the aromatic region of hIAPP in time in the absence (A) and in the presence of anle145c (B). The insert shows the integrated intensity of the peaks as function of aggregation time, corresponding to depletion of monomers and possibly small oligomers. The half-times of the depletion are rather similar. The peptide concentration is 50 μM and the anle145c concentration 10 μM. For further details see Materials and Methods section.

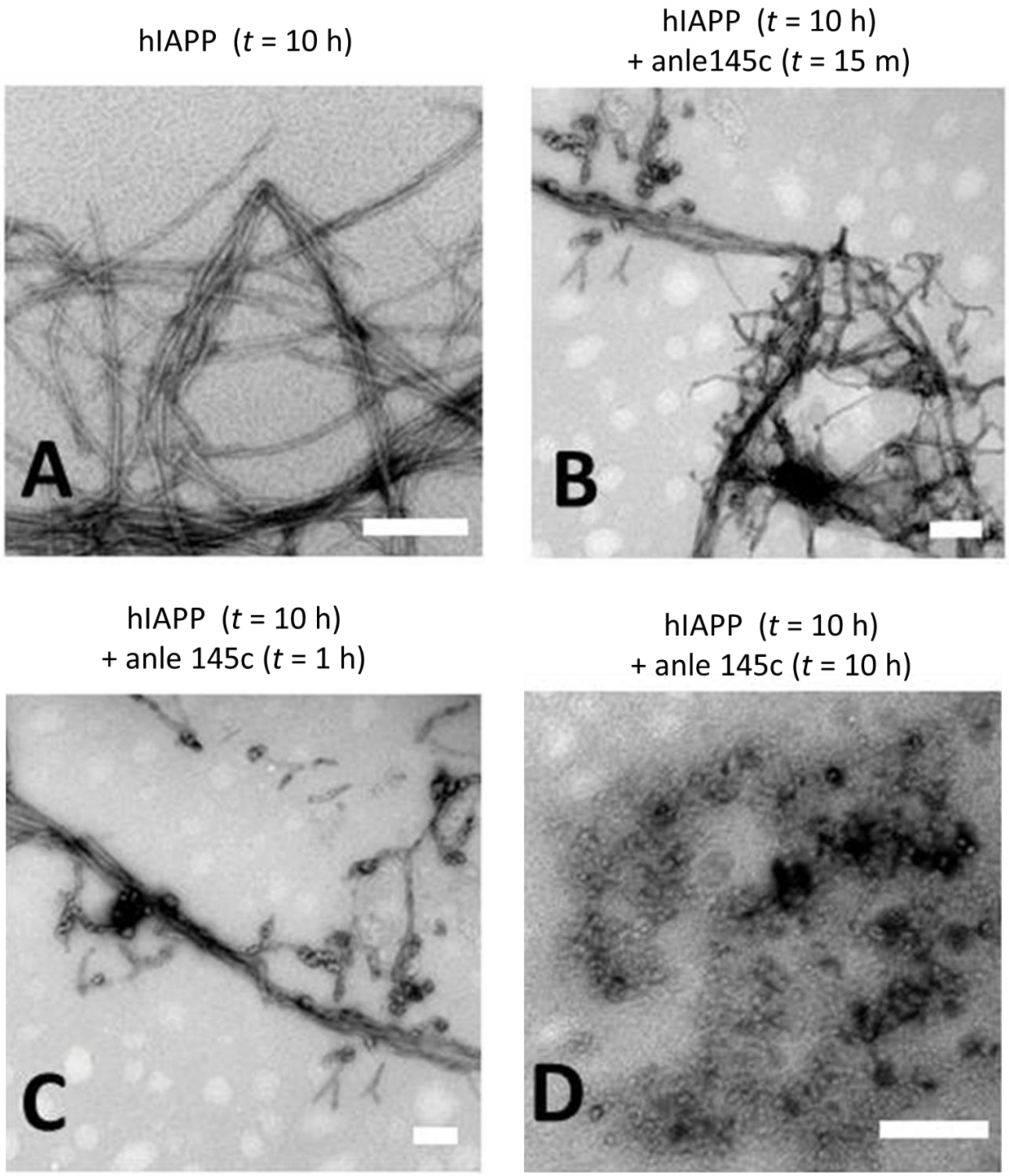

Figure S2. TEM images of hIAPP fibril formation after 10 h incubation (A) and effects of anle145c on the formed fibrils after different times of incubation: immediately after addition of anle145c (B), 1 h after addition of anle145c (C) and 10 h after addition of anle145c (D). (Scale bar represents 200 nm)

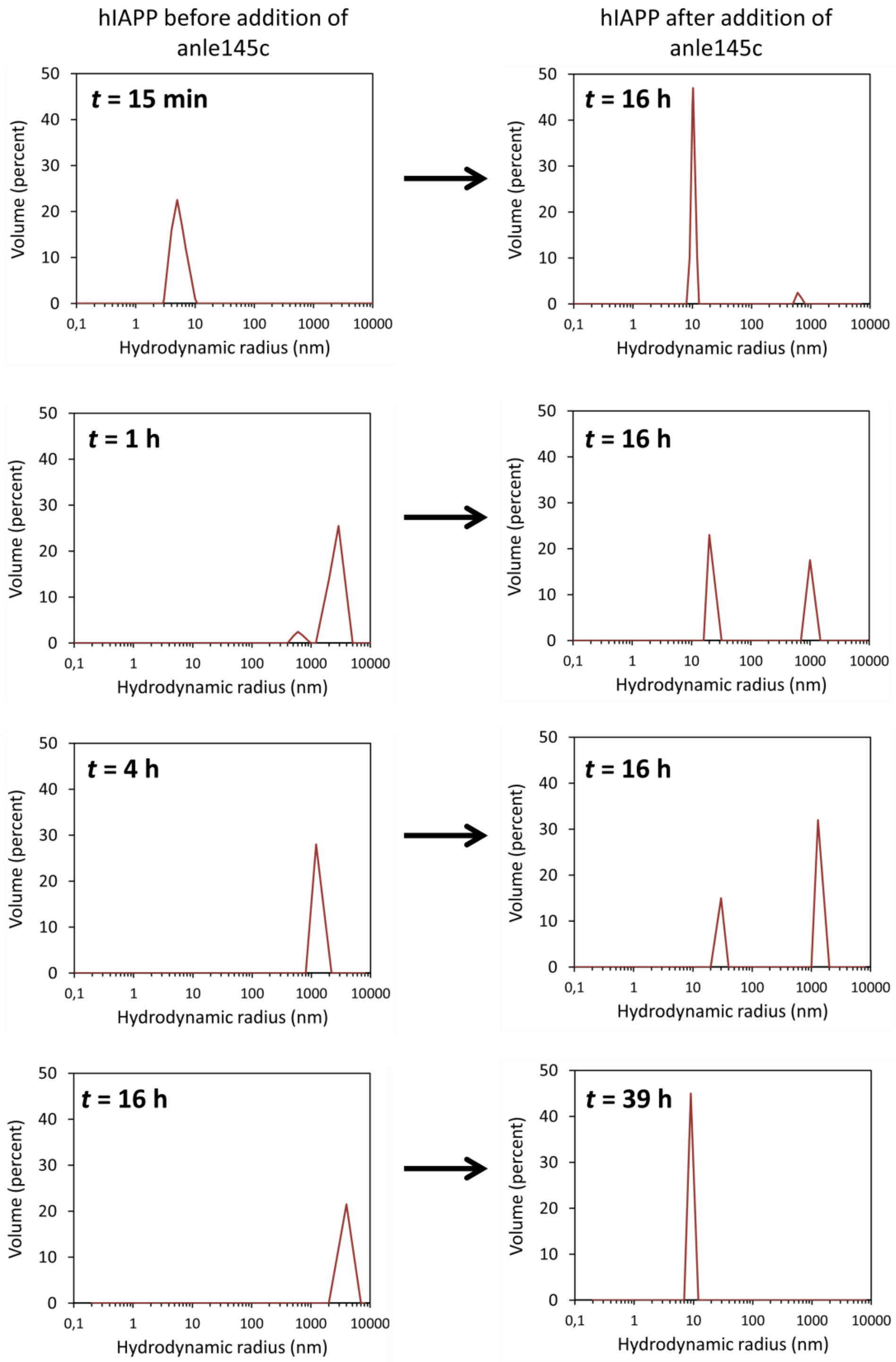

Figure S3. DLS measurements showing from top to bottom the size distributions of hIAPP amyloid species at different time points before (left panel) and after addition of anle145c (right panel). The time indicated in the right panel is the total incubation time of hIAPP.

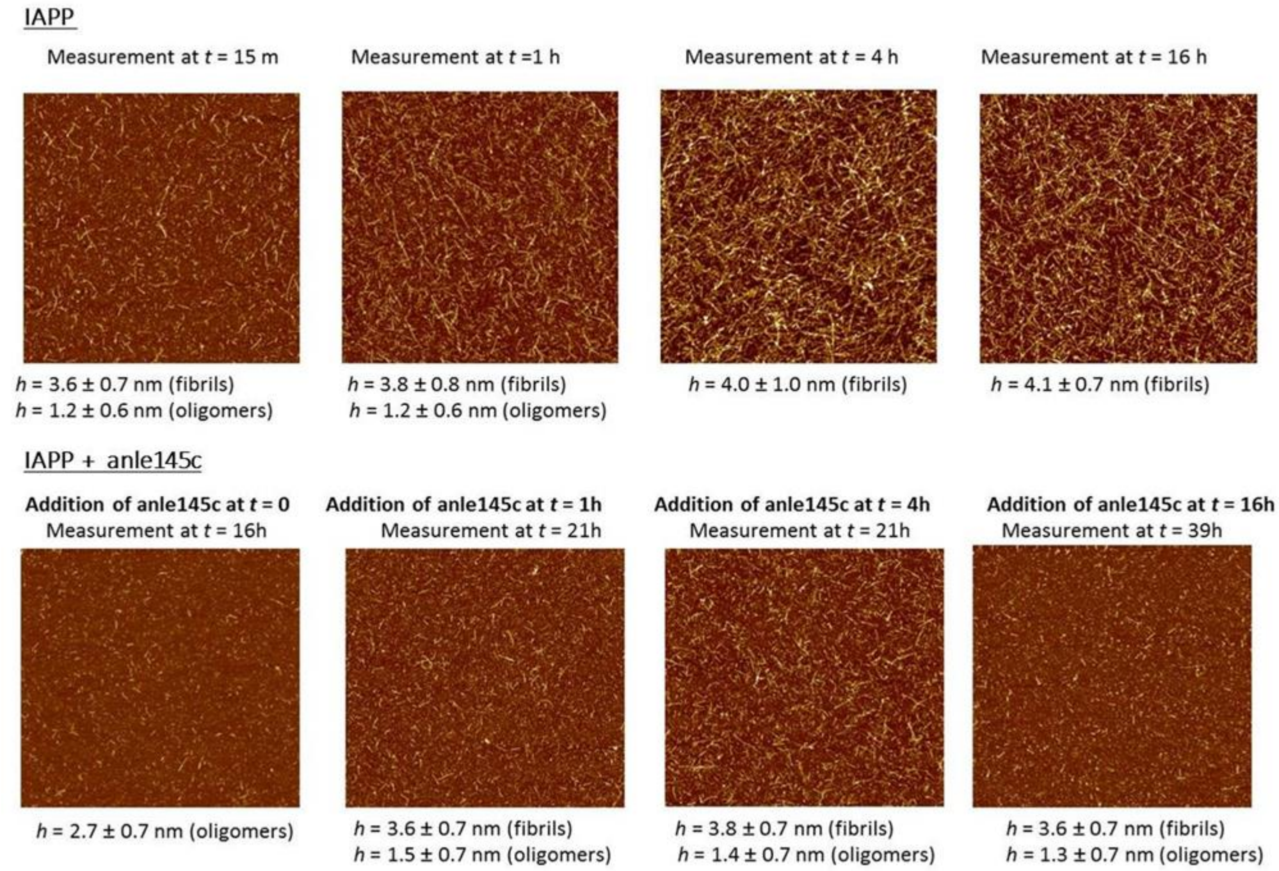

Figure S4. AFM micrographs of hIAPP amyloid species at different times of incubation (top) and after addition of anle145c to pre-incubated hIAPP amyloid species at the specified times (bottom).
